# Supplementary material for: Current-sensitive Hall effect in a chiral-orbital-current state
Source: Nat Commun. 2024 Apr 27;15:3579. doi: 10.1038/s41467-024-47823-2 (PMC11055857; doi:10.1038/s41467-024-47823-2)
Supplement: Supplementary file 1 — Supplementary Information [file 41467_2024_47823_MOESM1_ESM.pdf]

SUPPLEMENTARY FIGURES

Current-sensitive Hall effect in a chiral-orbital-current state

Yu Zhang[1], Yifei Ni[1], Pedro Schlottmann[2], Rahul Nandkishore[1,3] Lance E. DeLong[4], and Gang Cao[1,5*]

[1]*Department of Physics, University of Colorado at Boulder, Boulder, CO 80309, USA*

[2]*Department of Physics, Florida State University, Tallahassee, FL 32306, USA*

[3]*Center for Theory of Quantum Matter, University of Colorado at Boulder, Boulder, CO 80309, USA*

[4]*Department of Physics and Astronomy, University of Kentucky, Lexington, KY 40506, USA*

[5]*Center for Experiments on Quantum Materials, University of Colorado at Boulder, Boulder, CO 80309, USA*

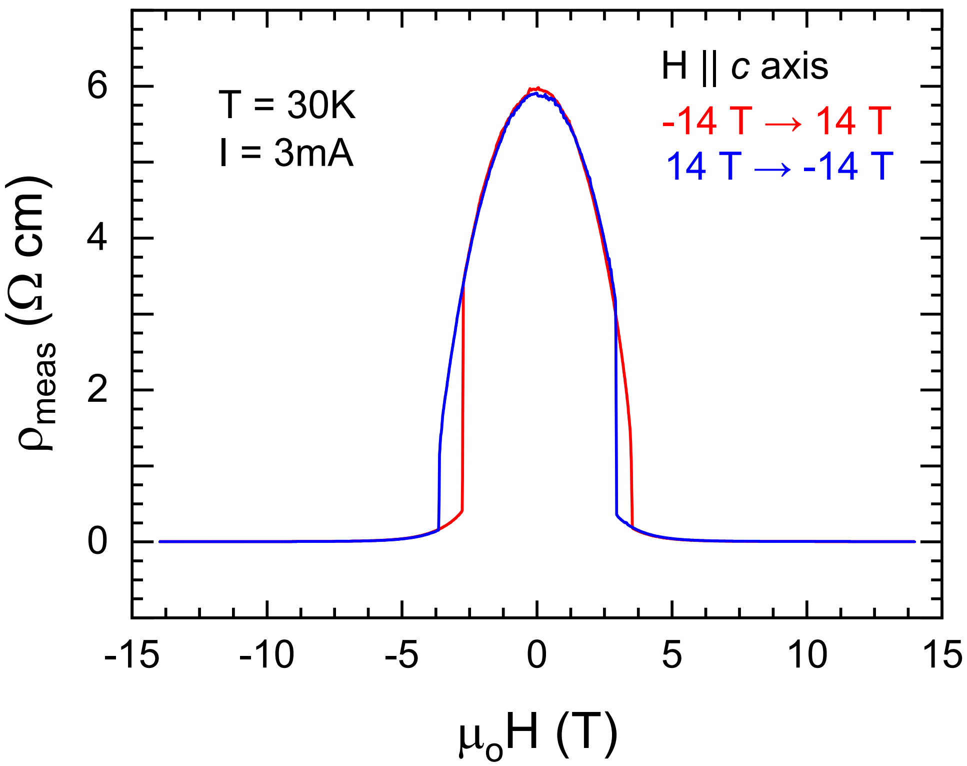

Supplementary Fig. 1

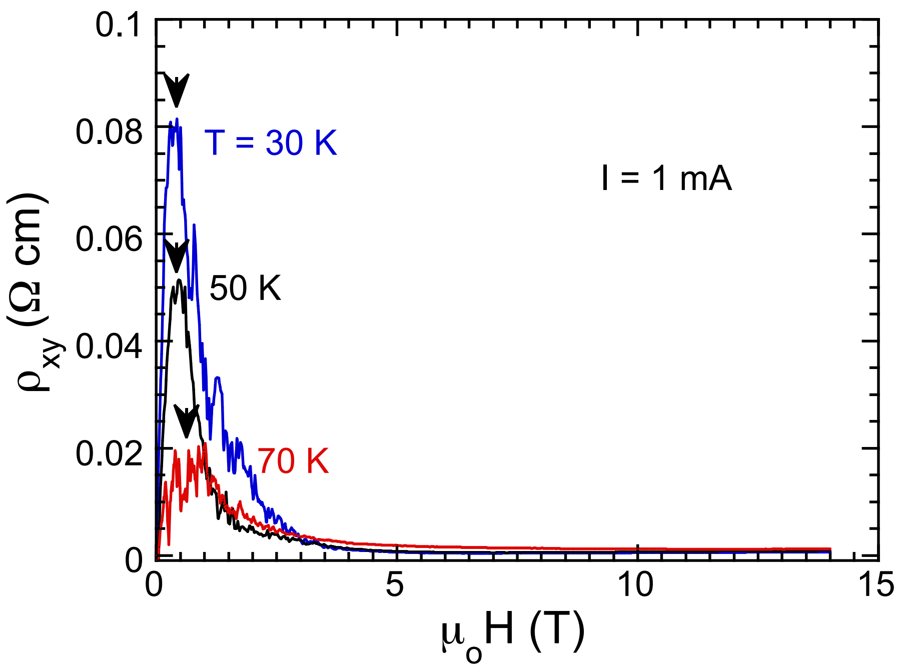

Supplementary Fig. 2

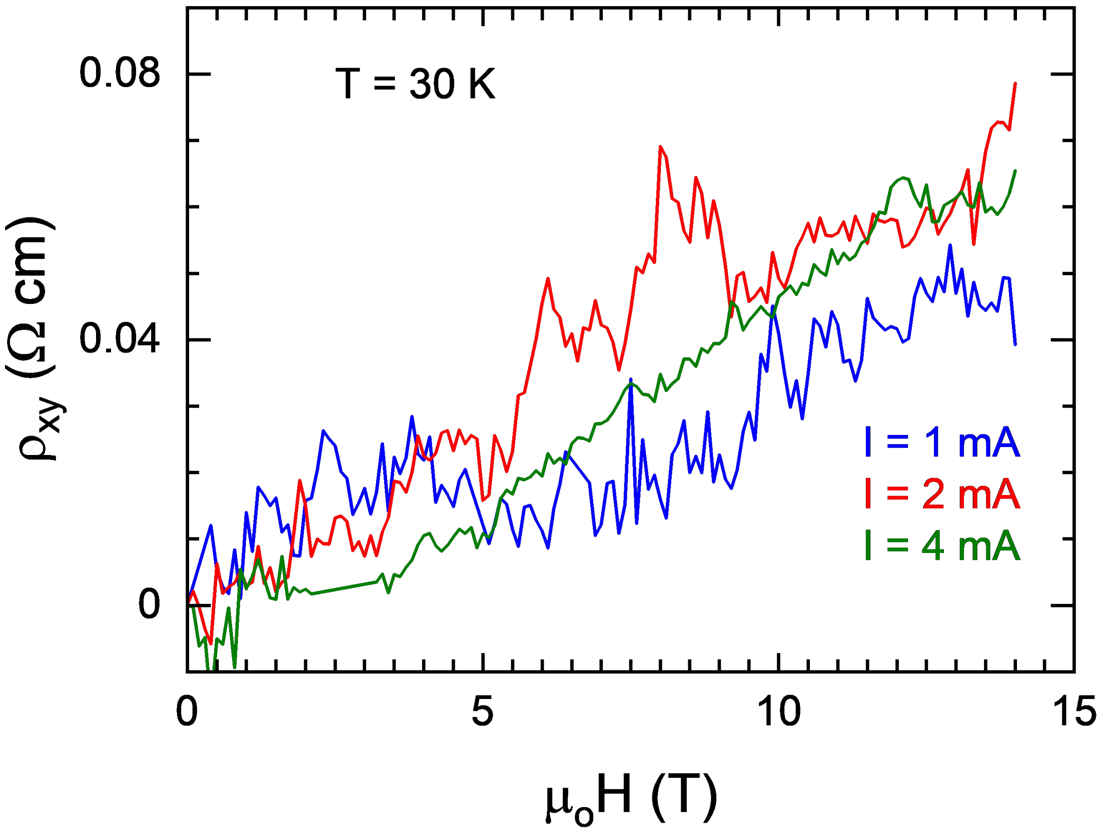

Supplementary Fig. 3

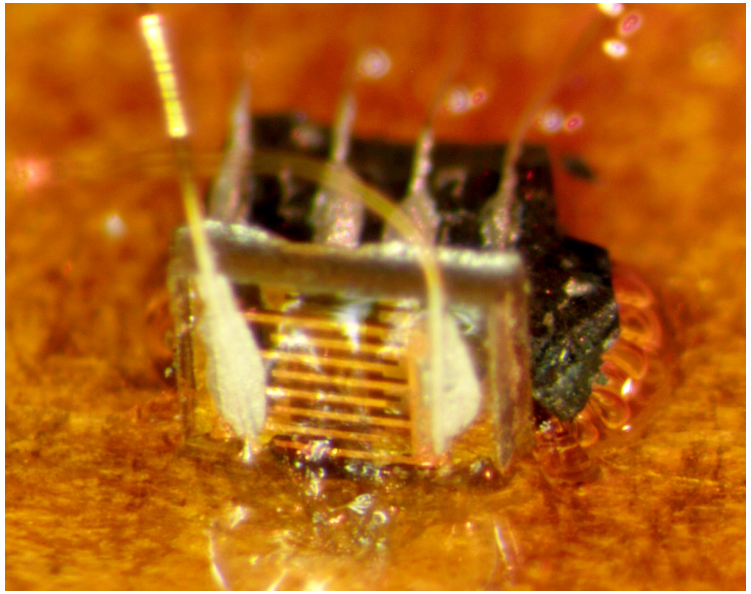

Supplementary Fig. 4

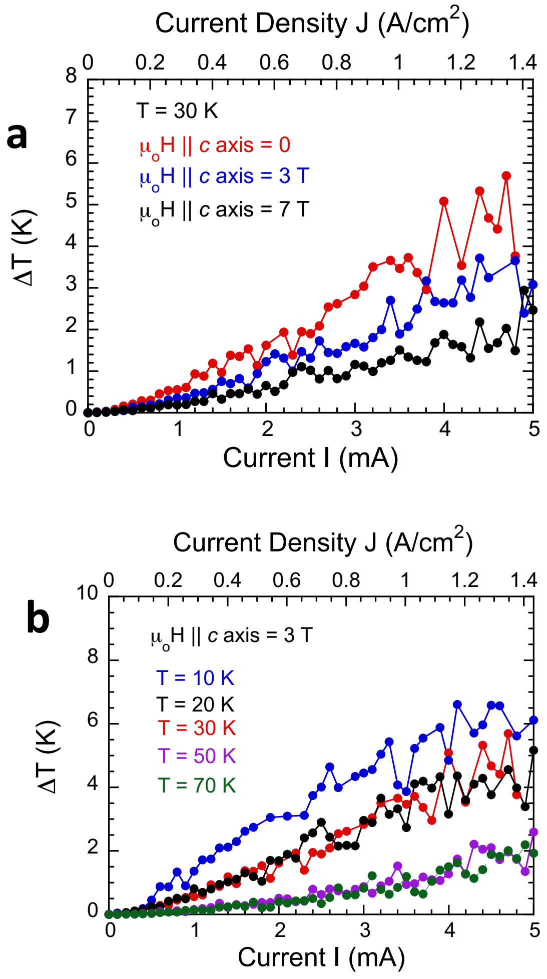

Supplementary Fig. 5

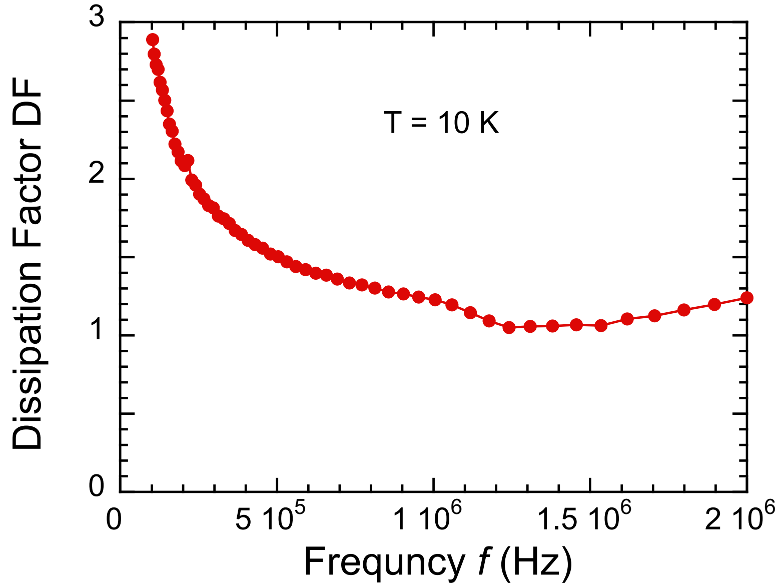

Supplementary Fig. 6

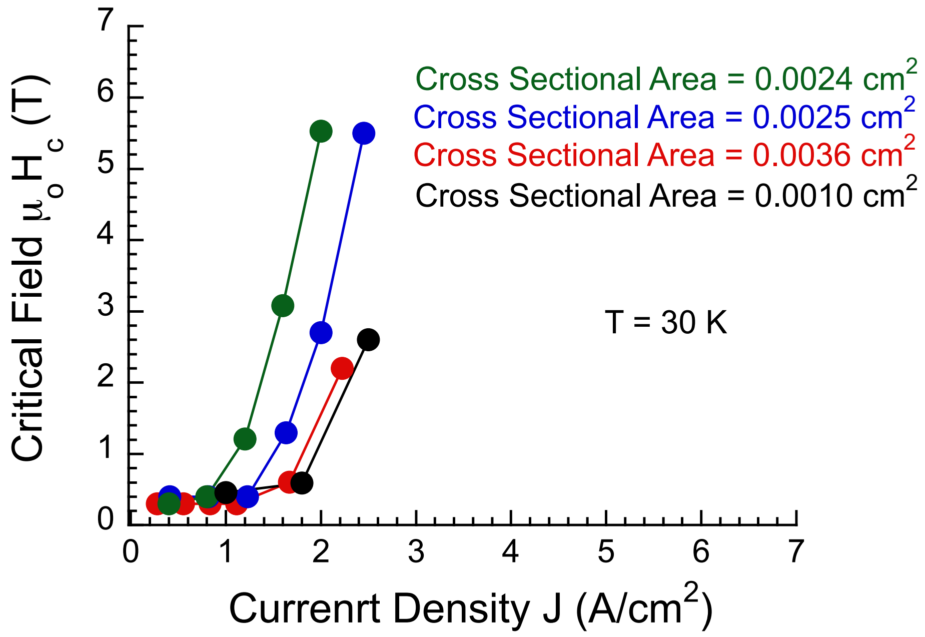

Supplementary Fig. 7

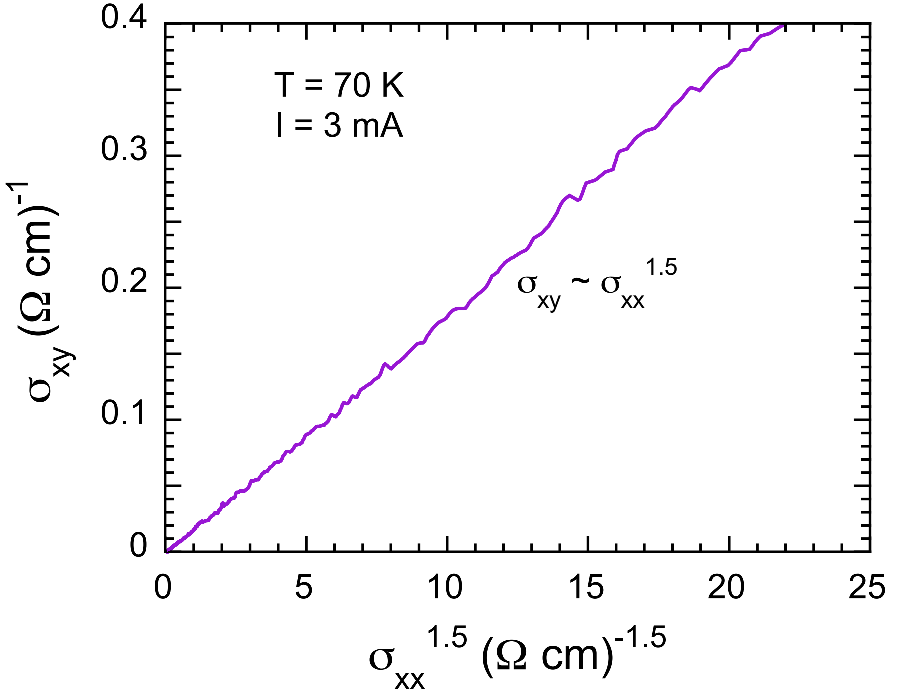

Supplementary Fig. 8

Supplementary Figures

Supplementary Fig.1. Hysteresis in the measured resistivity $\rho_{meas.}$ A representative set of data for ρ_{meas} at **H || *c*** axis from which ρ_{xy} and ρ_{xx} are obtained. Note the observed hysteresis is consistent with the formation of the COC domains discussed in the main text.

Supplementary Fig.2. The Hall effect as functions of magnetic field and temperature. The magnetic field dependence of ρ_{xy} (H||*c*) at I = 1 mA for selected temperatures. Note that the peak ρ_{xy}(H) at 1 mA remains essentially unshifted with increasing temperature, in contrast to the rapid shift of the peak with increasing external current I observed in ρ_{xy}(H) (Figs.2a-2e). The contrasting behaviors help rule out self-heating effects.

Supplementary Fig. 3. The Hall effect as functions of magnetic field and external current for H || *b* axis. The magnetic field dependence of ρ_{xy} (H||*b*) at T = 30 K for selected currents. Note the slope of ρ_{xy} (H||*b*) remains essentially unchanged, and there is no sign of AHE even though the *b* (or *a*) axis is the magnetic easy axis, where the magnetization is saturated at $\mu_o \mathbf{H}_{||b}$ > 0. 1 T.

Supplementary Fig. 4. Joule heating measurements. A Cernox thermometer (gold, front) is thermally contacted with a single-crystal sample of $Mn_3Si_2Te_6$ (black, behind the Cernox). The thin gold wires are electrical leads electrically attached to the sample and Cernox with an EPO-TEK H20E epoxy (silver). The light brown background is GE varnish used to thermally anchor the Cernox and the sample. Note that **H||c**-axis and I || a-axis.

Supplementary Fig. 5. Sample temperature change ΔT (K) as functions of applied current *I* (mA) and current density *J* (A/cm^2) a, at T = 30 K for representative magnetic fields m_oH || c axis, and **b**, at $\mu_o H_{||c}$ = 3 T for representative temperatures T.

Supplementary Fig. 6. Dissipation factor, DF, as a function of frequency f up to 2 MHz at T = 10 K and V = 1 V. The separation between the two electrodes is approximately 0.3 mm.

Supplementary Fig. 7. Critical Field $\mu_0 H_C$ as a function of current density *J* at $T = 30$ K for four different samples whose cross-sectional areas are displayed in the inset

Supplementary Fig. 8. Additional scaling relation. A scaling relation $\sigma_{xy} \propto \sigma_{xx}^{1.5}$ with $\alpha_{LF} = 1.5$ is observed at $T = 70$ K and $I = 3$ mA. Note that this behavior provides additional evidence that an external current exceeding a critical current can suppresses the COC state below T_C.
